# Molecular Typing of *Neisseria gonorrhoeae * Isolates by Opa-Typing and Ribotyping in New Delhi, India

**DOI:** 10.1155/2009/934823

**Published:** 2009-09-16

**Authors:** Pejvak Khaki, Preena Bhalla, Ahmad Mir Fayaz, Sohiela Moradi Bidhendi, Majid Esmailzadeh, Pawan Sharma

**Affiliations:** ^1^Microbiology Department, Razi Vaccine and Serum Research Institute, Karaj, Iran; ^2^Microbiology Department, Maulana Azad Medical College, New Delhi, India; ^3^D I-15, Rajouri Garden, New Delhi-110027, India; ^4^International Centre for Genetic Engineering and Biotechnology, New Delhi, India

## Abstract

Control and preventive measures for gonococcal infections are based on precise epidemiological characteristics of *N. gonorrhoeae* isolates. In the present study the potential utility of opa-typing and ribotyping for molecular epidemiological study of consecutive gonococcal strains was determined. Sixty gonococcal isolates were subjected to ribotyping with two restriction enzymes, *Ava*II and *Hinc*II, and opa-typing with *Taq*I and *Hpa*II for epidemiological characterization of gonococcal population. Ribotyping with *Ava*II yielded 6 ribotype patterns while twelve RFLP patterns were observed with *Hinc*II. Opa-typing of the 60 isolates revealed a total 54 opa-types, which 48 were unique and 6 formed clusters. Fifty-two opa-types were observed with *Taq*I-digested PCR product while opa-typing with *Hpa*II demonstrated 54 opa-types. The opa-types from isolates that were epidemiologically unrelated were distinct, whereas those from the sexual contacts were identical. The results showed that opa-typing is highly useful for characterizing gonococcal strains from sexual contacts and has more discriminatory than ribotyping that could differentiate between gonococci of the same ribotype. The technique even with a single restriction enzyme has a high level of discrimination (99.9%) between epidemiologically unrelated isolates. In conclusion, the molecular methods such as opa-typing and ribotyping can be used for epidemiological characterization of gonococcal strains.

## 1. Introduction

*Neisseria gonorrhoeae* is the causative agent of gonorrhea, which continues to be one the most common sexually transmitted infections worldwide [1, 2]. Control and preventive measures for infection are based on epidemiological analyses as well as antibiotic susceptibility of the *N. gonorrhoeae* isolates. The process of typing of *Neisseria gonorrhoeae* isolates can be used to identify the population of strains that circulate in different communities, temporal, and geographic changes among the strains, transmission patterns, and antibiotic resistance outbreaks [3, 4].

Various typing methods have been developed to characterize *N. gonorrhoeae *isolates. The widely used phenotypic methods are based on auxotyping, serotyping, and antimicrobial susceptibility testing. However, these methods have some limitations such as reproducibility and discriminatory power [5–9]. Therefore, different molecular genetic methods have been developed for the characterization of gonococcal strains include ribotyping [10, 11], pulsed-field gel electrophoresis (PFGE) [12], arbitrarily primed PCR [13], Amplified Fragment length polymorphism (AFLP) [14], opa-typing [15, 16], and sequence typing based on one or more genes [4, 13, 16, 17].

Ribotyping refers to the use of Southern blot analysis for detecting polymorphisms that are associated with the ribosomal DNA regions. The method has appeared to be good reproducibility, stability, and typeability for characterization of gonococcal isolates. However, it provides less discrimination between isolates of *N. gonorrhoeae* [10, 11].

Opa-typing is a PCR-based RFLP technique, which relies on amplifying the 11 *opa* genes and subsequent digestion with restriction enzymes, to generate restriction fragment length polymorphism. The method has shown to be highly discriminatory enough to be applied to large collections of gonococcal isolates [15]. The opa-typing is also useful to confirm self-reported sexual contacts and to identify sex partners and short chains of transmission [15, 16, 18].

There are hardly any data about molecular epidemiological analysis of *N. gonorrhoeae* isolates by opa-typing and ribotyping in India. Epidemiological analysis of *N. gonorrhoeae* isolates by genotypic methods has not been carried out in India so far.

In this study, we determined the potential utility of opa-typing and ribotyping for differentiation of gonococcal strains.

## 2. Material and Methods

### 2.1. Gonococcal Isolates and Culture Conditions

Sixty consecutive *N. gonorrhoeae* strains isolated from among 62 males with urethritis, 22 females with endocervicitis, and 10 sexual contacts of these patients attending the STD clinic of Lok Nayak Hospital, New Delhi from January, 2006 to June, 2007 were studied. All gonococcal isolates were subjected to opa-typing and ribotyping for epidemiological characterization.

The samples were inoculated directly onto the selective Modified Thayer-Martin medium (MTM) medium and incubated at 35–36.5°C containing 5%CO2 for 48 hours. The colonies suspected to be *N. gonorrhoeae* were identified by gram stain, oxidase, superoxol tests, and rapid carbohydrate utilization test (RCUT). Gonococcal isolates were stored at −70°C [19].

### 2.2. Isolation of Genomic DNA

Genomic DNA was isolated from confluent (18–24 hours) growth of gonococcal cells by using a Wizard genomic DNA purification kit (Promega Corp., Madison, USA) according to the manufacturer's instructions. Briefly, the bacterial cell was suspended in 500 *μ*L of Tris-EDTA buffer (pH 7.8). The suspensions were pelleted and resuspended in 500 *μ*L of Nuclei lysis solution (Promega) and incubated for 5 minutes at 80°C. However, 4 *μ*L of RNase (10 mg/mL) (Sigma Chemical Co., USA) was added to cell lysate and incubated for 1 hour at 37°C. The lysate was adjusted to 0.7 M NaCl and 1% cetyltrimethylammonium bromide (CTAB), mixed thoroughly, and incubated for 10 minutes at 65°C in order to precipitate cell wall debris, denatured proteins, and polysaccharides. The supernatant was extracted twice with phenol/chloroform/isoamyl alcohol (25 : 24 : 1) and twice with chloroform/isoamyl alcohol (24 : 1). Chromosomal DNA was precipitated with isopropanol, washed with 70% ethanol, resuspended in 50 *μ*L of Tris-EDTA buffer (pH 7.8), and stored at −20°C.

### 2.3. Ribotyping

Chromosomal DNA was digested with *Ava*II and *Hinc*II, and the restricted fragments were separated by electrophoresis on 0.8% agarose gels in Tris-borate (TB) buffer at 40 V for 16 hours. DNA fragments were transferred to positively charged nylon membrane (Roche Diagnostic GmbH, Mannheim, Germany) from agarose gels by the method of Southern. The blot was hybridized with the addition of digoxigenin-dUTP labeled cDNA probe at 68°C for 18–24 hours [20].

However, 16S and 23S rRNA from *E*. *coli* (Roche Diagnostic GmbH, Mannheim, Germany) was reverse transcribed into cDNA with SuperScript II reverse transcriptase (Invitrogen, Calif, USA). The cDNA was then labeled by random priming with digoxigenin-dUTP according to the manufacturer's directions (Roche Diagnostic GmbH, Mannheim, Germany).

The sizes of the hybridized fragments from at least four blots were averaged after measuring distances of band migration in comparison with the Digoxigenin labeled DNA molecular weight marker III (Roche Diagnostic GmbH, Mannheim, Germany).

### 2.4. Opa-Typing

Opa-typing was performed as described previously [16]. Briefly, opa genes were amplified from gonococcal chromosomal DNA by PCR. Amplification was carried out in a 50 *μ*L volume containing 0.5 *μ*g of each opa forward and reverse primer (opa-01, 5′-ATGTCAGGCGGATTTAGCC-3′, and opa-04, 5′-AATGAGGCTTCGTGGGTTTTG-3′), 100 ng of chromosomal DNA, 0.2 mM of each dNTP, 2.5 U of Taq DNA polymerase, and the buffer provided by the manufacturer with 75 mM Tris HCl (pH 9.0), 2 mM MgCl_2_, 50 mM KCl, 20 mM (NH_4_)_2_SO_4_ (Biotools, B&M Labs, S.A., Madrid, Spain). Cycle conditions were set at 94°C for 5 minutes (hot-start); 30 cycles of 94°C for 1 minute, 64°C for 1 minute, and 72°C for 1 minute; a final extension reaction at 72°C for 10 minutes. The PCR products (≈670 bp) were precipitated with ethanol, washed with 70% ethanol, and resuspended in 50 *μ*L of TE buffer. The *opa* gene fragments (1.5 *μ*g) were digested overnight at 65°C with 10 U of *Taq*I in a volume of 30 *μ*L using the buffer recommended by manufacturer. An equivalent aliquot was digested overnight at 37°Cwith 10 U of *Hpa *II. The resulting restriction fragments was fractionated on a precast 12% polyacrylamide gel and visualized by ethidium bromide staining.

### 2.5. Analysis of the Typing Methods

The reproducibility of ribotyping and opa-typing was examined by analysis of the same bacterial strain on three separate occasions. The stability of the techniques was examined by daily subcultures of *N. gonorrhoeae* isolates. The discriminatory index for two methods was calculated as described previously [21]. The relatedness values were calculated as the proportion of unweighted mismatches of fragments between the different patterns. Computed similarity cluster analysis of the patterns was done by the unwieghted pair-group average algorithm, using the Dice coefficient to analyze the similarities of the banding patterns. The phylogenetic tree (dendogram) was constructed by DNASTAR software package.

### 2.6. Statistical Analysis

Statistical methods like Chi-square test and Fisher's exact test were performed to determine the significant association of various demographic data using the SPSS version 13.0 for Windows.

## 3. Results

A total of 60 gonococcal strains were isolated from 52 (83.87%) out of 62 men with urethritis, 4 (18.18%) out of 22 women with endocervicitis, and 4 (40%) out of 10 sexual contacts of these cases. All gonococcal isolates were subjected to opa-typing and ribotyping for epidemiological characterization.

### 3.1. Ribotyping

**Figure 1:**
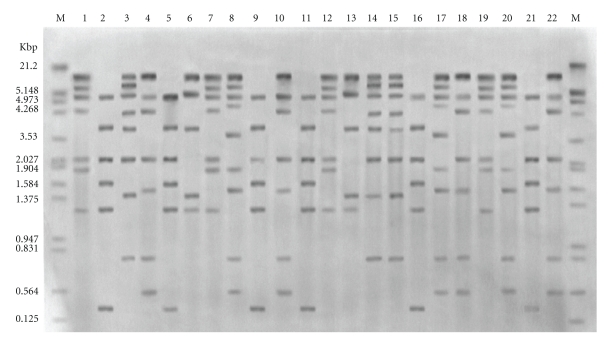
Examples of *Hinc*II ribotype patterns of consecutive gonococcal isolates: lane M: digoxigenin labeled DNA molecular weight marker III (Roche Diagnostics Corp., Indianapolis, USA); lanes: 1, 7, 12, and 19: ribotype pattern RH6; lanes: 2, 5, 9, 11, 16, and 21: ribotype pattern RH1; lanes: 3, 14, and 5: ribotype pattern RH9; lanes: 4, 10, 18, and 22: ribotype pattern RH3; lanes: 6 and 13: ribotype pattern RH11; lanes: 8, 17, and 20: ribotype pattern RH7.

Chromosomal DNAs from 60 isolates were digested with *Hinc*II and *Ava*II. Hybridization of *Ava*II fragments with labeled rRNA probes yielded 6 ribotype patterns (Table 1). Twenty-three of the 60 isolates demonstrated pattern 1; 16 isolates had patterns 2; 12 isolates demonstrated pattern 3; 4 isolates had pattern 4; 3 isolates pattern 5; 2 isolates pattern 6. Analysis with *Hinc*II produced highly resolved fragments suitable for analysis, and fragment sizes ranged from 0.46 to 18.36 kb (Figure 1). Twelve RFLP patterns were observed with *Hinc*II, from which 16 of the 60 isolates demonstrated pattern 1; 10 isolates had pattern 2; 8 isolates had pattern 3; patterns 4–6 comprised 4 isolates each; patterns 7–9 contained 3 isolates each; patterns 10 and 11 had 2 isolates each. One isolate demonstrated pattern 12. All isolates recovered from index case and their sexual contacts were found to be identical by ribotyping.

The characteristic isolate banding patterns with *Hinc*II remained unchanged when DNA was prepared from randomly selected strains grown on separate occasions, and the ribotypes did not change after three individual strains were subcultured 1, 10, and 20 times.

### 3.2. Opa-Typing

**Figure 2:**
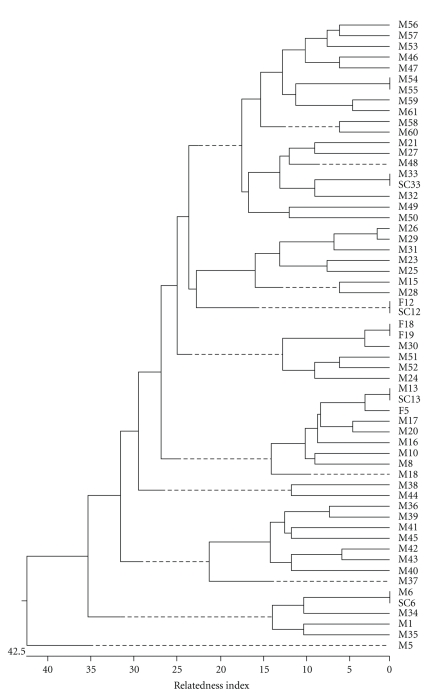
Dendogram of the cluster analysis of the 60 consecutive gonococcal isolates obtained after opa-typing with *Hpa*II. The relatedness values were calculated as the proportion of unweighted mismatches of fragments between the different patterns. The dendogram was generated by DNASTAR software package.

**Figure 3:**
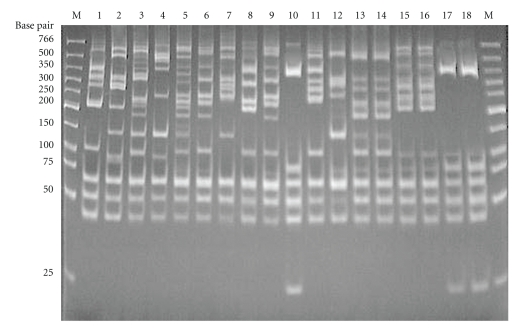
Examples of different patterns of *Hpa*II opa-types of consecutive gonococcal isolates. Lane M: molecular weight marker (New England Biolabs, USA); lanes 1–18: *N. gonorrhoeae* isolates; sexual contacts: lanes 13, 14 (HpaII opa-type 13), lanes 15, 16 (HpaII opa-type 14), lanes 17, 18 (HpaII opa-type 15).

For opa-typing, the 11 *opa* genes were amplified and the PCR products were digested with restriction enzymes *Taq*I and *Hpa*II. Dendogram obtained from opa-typing with *Hpa*II is shown in Figure 2. Cluster analysis of *Hpa*II opa-type patterns revealed a total 54 opa-types, from which 48 (88.9%) were unique and 6 (11.1%) formed clusters (Table 1 and Figure 2). Of the 48 isolates with unique opa-types, none were known to be from sexual contacts. Fifty-two opa-types were observed with *Taq*I-digested PCR product. Forty-five had unique opa-type while 7 formed clusters. Opa-typing with *Taq*I was slightly less discriminatory than typing with *Hpa*II (Table 1). The opa-types from isolates that were epidemiologically unrelated (unconnected) were distinct, whereas those from the sexual contacts were identical. Isolates M54 and M55 could not be distinguished by both enzymes. Examples of the *Hpa*II opa-type patterns are given in Figure 3.

PCR amplifications of the *opa* genes were performed from three isolates of *N. gonorrhoeae* grown on separate occasions, from which the opa-types were identical. The stability of the opa-typing during subculture in vitro was examined by obtaining opa-types from DNA prepared from daily subcultures of three isolates of *N. gonorrhoeae.* No differences in the opa-types of the isolates were seen after 1, 10, and 20 subcultures.

## 4. Discusion

The ability to identify accurately the strains of infectious agents that cause disease is central to epidemiological surveillance and public health decisions. The widely available typing systems have the potential to provide information useful to the control and prevention of *N. gonorrhoeae* infections and to guide health interventions. All typing systems for epidemiological studies should have established reproducibility, and be highly discriminating in order to distinguish epidemiologically unrelated infections. The shortcomings of phenotypic typing methods have led to development of molecular genetic methods, which minimize problems with reproducibility or discriminatory power [5–9, 12, 15, 16].

There is hardly any data about epidemiological studies of *N. gonorrhoeae* isolates based on molecular typing in India. Therefore, our aim was to assess molecular typing of *N. gonorrhoeae* isolates using ribotyping and opa-typing.

Ribotyping has the advantage over restriction endonuclease analysis in that the RFLP patterns contain fewer bands for comparison because only fragments with rRNA sequences are visualized. More pronounced heterogeneity of rRNA hybridization patterns could be generated by restriction endonuclease that have different restriction sites within the rRNA genes. In our study, RFLP patterns generated with *Hinc*II (12 ribotype patterns) showed greater heterogeneity than those generated by *Ava*II (6 ribotype patterns). On the basis of combined *Hinc*II and *Ava*II-generated RFLP patterns, 60 isolates of *N. gonorrhoeae* were classified into 16 ribotype patterns, and no isolate was nontypeable. The results of this study showed that ribotyping was much simpler to interpret than the complex restriction endonuclease patterns of total chromosomal DNA. The technique was also found to be both stable and reproducible. The results suggested that there may be sufficient heterogeneity of rRNA patterns within *N. gonorrhoeae* isolates and the method may to be useful for differentiation of strains in epidemiological studies.

A similar study has shown that RFLP patterns generated by *Hinc*II could be used to differentiate 43 isolates of *N. gonorrhoeae* into nine patterns [22]. A previous study of ribotype patterns generated by combination of *Sma*I and *Ava*II digests of *N. gonorrhoeae* differentiated 23 proline-citrulline-uracil requiring (PCU^−^) auxotypes into four groups [10]. A Canadian study indicated that the ribotyping by *Sma*I could be classified 27 ornithine-uracil-hypoxanthine (OUH^−^) or citrulline-uracil-hypoxanthine (CUH^−^) or ornithine-hypoxanthine (OH^−^)/IA-2 auxotypes of *N. gonorrhoeae* into five ribotype patterns [11]. The present study showed that common DNA bands were present in the RFLP patterns for all the *N. gonorrhoeae* isolates in accordance with previous studies [10, 11, 22].

Opa-typing is a technique based on diversity (polymorphism) of *opa* genes of *N. gonorrhoeae*. A single gonococcus possesses 11 different *opa* genes, which encodes the outer membrane proteins. The extensive variation and rapid evolution of the *opa* gene repertoire make it likely that gonococci with identical opa-types are very closely related genetically and epidemiologically. Major potential use of opa-typing is the identification of groups of individuals in sexual networks by the isolation of gonococci with the same opa-type from gonococcal populations recovered in a restricted geographic area within a limited time period [15].

In this study, opa-typing of the 60 isolates revealed a total of 54 opa-types, from which 48 (88.9%) were represented by single isolates. The opa-types from isolates that were epidemiologically unrelated were distinct, while those from the sexual contacts were identical. There were only two consecutive isolates being indistinguishable by opa-typing. As the gonococci within these were indistinguishable by ribotyping, and were from patients living in the same part of Delhi attending the STD clinic within a short time period, it is possible that they were from individuals who were connected by a short chain of infection transmission or were form common core group (sexual contacts). This discrepancy was also found in a previous study [15].

Opa-typing identified all of the isolates from sexual contacts. This result indicates that opa-typing is highly useful for characterizing gonococcal strains from sexual contacts in full agreement with previous studies [15, 16, 18].

We found opa-typing to be more discriminatory than ribotyping and could differentiate between gonococci of the same ribotype. The technique even with a single restriction enzyme (*Hpa*II) has a high level of discrimination (99.7%) between epidemiologically unrelated isolates in concordance with results in previous studies [15, 16, 18, 23].

The results of both methods indicate that gonococcal infection among the patients studied was not derived from common core groups. In conclusion, the molecular methods such as opa-typing and ribotyping can be used for epidemiological characterization of gonococcal strains.

## Figures and Tables

**Table 1:** Discriminatory indices of typing methods for gonococcal isolates.

Typing method	number of type patterns	Discriminatory index
Ribotyping		
With *Ava*II	6	0.75
With *Hinc*II	12	0.87

Opa-typing		
With *Taq *I	52	0.995
With *Hpa *II	54	0.997
